# Boosters and Barriers to Implementation: Youth‐Initiated Mentoring for Justice‐Involved Youth

**DOI:** 10.1002/jcop.70051

**Published:** 2025-10-26

**Authors:** Angelique Boering, Annabeth P. Groenman, Levi van Dam, Geertjan Overbeek

**Affiliations:** ^1^ University of Amsterdam Amsterdam The Netherlands; ^2^ Levvel Academic Centre for Child and Adolescent Psychiatry Amsterdam The Netherland

**Keywords:** diversion programme, focus groups, justice‐involved youth, programme implementation, programme staff, youth‐initiated mentoring

## Abstract

Recently, it has been argued that youth‐initiated mentoring (YIM) holds promise for justice‐involved youth. In YIM, youths select an adult mentor from their social network. Successful implementation is key to the effectiveness of an innovation, but little is known about the factors contributing to the successful implementation of YIM. We explored boosters and barriers to implementation perceived by professionals implementing YIM into a juvenile diversion programme. We performed thematic analysis on one unstructured (*N* = 22) and three semi‐structured focus groups (*N* = 7–8) guided by the Consolidated Framework for Implementation Research. Findings suggest that professionals perceived the benefits of YIM in this context. Nevertheless, the current organisational culture, grounded in *Risk‐Need‐Responsivity* principles, hindered the actual implementation in certain cases, possibly endangering the continuation of YIM in a juvenile diversion context. Boosters and barriers can be considered and adopted by organisations aiming to implement YIM in a selective prevention context.

**Trial registration number:** (ClinicalTrial.gov ID #NCT05555472).

Youth mentoring is a widely applied preventive strategy for youth at risk of and engaging in delinquent behaviour (Tolan et al. 2013). Recently, it has been argued that, specifically, *informal* or *natural* mentoring approaches, such as Youth‐Initiated Mentoring (YIM), hold promise in promoting positive outcomes for these youths (Chan and Henry 2005; Spencer et al. 2019). In YIM, youth choose a non‐parental adult as a mentor (Schwartz et al. 2013; Van Dam and Schwartz 2020). This approach seems particularly suitable for justice‐involved youth since they are often more reluctant to accept help from an unknown mentor and are thus challenging to match with a mentor via traditional approaches (Spencer et al. 2019). Over the last decade, YIM has been implemented in various contexts, such as youth care organisations or juvenile justice system organisations (Koper et al. 2020; Schwartz et al. 2013; Dam et al. 2017). Evidence on the effect of natural mentoring programmes, such as YIM, on delinquency is mixed. Three American studies have focused on at‐risk youths, specifically those who had dropped out of or were expelled from high school, but not necessarily justice‐involved. A randomised trial evaluating a programme with a natural mentoring component found decreased involvement in delinquency at the 21‐month follow‐up (Millenky et al. 2010), although this effect was not observed at the study's 36‐month follow‐up (Millenky et al. 2011). However, a non‐randomised follow‐up using the same study sample, focusing solely on the natural mentoring component, reported an association between those still in contact with their natural mentors and decreased delinquency involvement at 38 months (Schwartz et al. 2013). In three Dutch randomised trials on justice‐involved youth, there were no effects of natural mentoring programmes on delinquency outcomes (De Vries et al. 2017, 2018; James et al. 2016). A possible explanation for these mixed results can be found in the different implementation levels of YIM across studies. Hence, it is crucial to examine the implementation of YIM. In this study, we aimed to gain insight into the boosters and barriers experienced by professionals working with YIM in a short‐term diversion programme for justice‐involved youth.

## 1 Youth‐Initiated Mentoring Implemented for Justice‐Involved Youth

In YIM, the choice of a mentor lies with the youth (Ruig and Van Dam 2021; Schwartz et al. 2013; Van Dam and Verhulst 2016). Youths ask someone they already know and with whom a relationship naturally exists, such as an aunt or a neighbour, to be their YIM. This contrasts with formal mentoring approaches, in which professionals seek a match between a youth and an adult volunteer, usually in the context of a school or community programme (Raposa et al. 2019). Professionals implementing YIM support youth in searching for and choosing a YIM and work together closely with all parties (i.e., youths, caregivers, and YIMs). The YIM is a trustee and spokesperson for youths, a partner to caregivers, and closely involved in decision‐making alongside professionals. Most importantly, the approach integrates support from the informal social network and the formal support system, capitalising on each other's strengths to support the youth (Ruig and Van Dam 2021; Van Dam and Verhulst 2016).

Recently, YIM has been implemented as an addition to a short‐term diversion programme in the Netherlands aimed at strengthening resilience and preventing reoffending in justice‐involved youth. Halt, a Dutch juvenile justice system organisation, has the nationwide task to provide this tailored diversion programme to 12–18‐year‐old youths after committing light offences, such as violations of the educational law (truancy), underage drinking, shoplifting, or vandalism (www.halt.nl). In their programme, youths – mainly first‐ and second‐time offenders – learn from their mistakes by gaining insight into the consequences of their behaviour and making amends with their victims. Moreover, Halt focuses on strengthening resilience by teaching youths how to deal with setbacks, set boundaries, and recognise negative influences to resist them. If youths complete the diversion programme, the offence is not reported on their criminal record.

The programme follows the principles of the *Risk‐Need‐Responsivity* (RNR) model (Bonta and Andrews 2007). In line with this model, professionals at Halt match the intensity of the programme to the youths' *risk* of reoffending, specifically assess and target the youths' *needs*, and focus on the youths' *responsivity* by tailoring the programme to the youths' capabilities, strengths, and motivation. For example, adapting the programme to youths' *needs* might entail that youths involved in an offence due to peer pressure receive support on building resistance to peer influences, while youths who have difficulties with authority receive support on dealing with authority. When more *risks* are identified, professionals intensify the programme (or vice versa with no or few risks) or refer these vulnerable youth to necessary support systems, such as psychologists or addiction treatment centres. However, in practice, professionals observed that youths (and their families) were quite often reluctant towards professional support or that problems were not ‘complex’ or ‘heavy’ enough for this type of support. Importantly, this is where YIM implementation comes in: offering professionals an option to strengthen the youths' social network and provide them with additional support, if necessary.

## 2 Implementation Research on Youth‐Initiated Mentoring

Correct implementation of an intervention is crucial to accurately interpret intervention outcomes (Durlak and DuPre 2008). Moreover, knowledge of implementation allows for detecting implementation problems in an early phase and correcting them, and it stimulates the actual and proper use of an intervention (Durlak and DuPre 2008; Fleuren et al. 2014). Currently, however, very little is known about the extent to which, or how, YIM is implemented for justice‐involved youths.

To our knowledge, one previous study focused on the boosters and barriers experienced by professionals while implementing YIM in child welfare and juvenile justice systems (Spencer et al. 2021). This study showed that, on the one hand, professionals experienced barriers in the identification process of YIM, such as concerns and difficulties identifying a YIM, discouragement of professionals due to the difficulty for youths (and their caregivers) in identifying a potential mentor, and a larger time investment compared to formal matching procedures. On the other hand, professionals experienced boosters once a YIM was successfully identified, such as less intensive guidance after matching due to a strong connection developing more rapidly and a more durable relationship than in formal mentor relationships (Spencer et al. 2021).

While the study by Spencer et al. (2021) focused partly on the professionals' perspectives in the juvenile justice system, it did not use a formal theory to map the boosters and barriers of implementation. However, within implementation research, there is a strong urge to use theoretical frameworks, as it has several advantages: a theoretical framework can guide data collection and the analysis and interpretation of one's findings, and it helps to generalise the findings across studies and domains (Eccles et al. 2009; Kirk et al. 2015). Therefore, the present study developed an interview protocol and codebook to assess professionals' perceptions of boosters and barriers when implementing YIM, based on the Consolidated Framework for Implementation Research (CFIR) (Damschroder et al. 2009, 2022).

The CFIR is among the most applied frameworks aiming to assess determinants (i.e. boosters and barriers) that impact implementation outcomes (Damschroder et al. 2022). The CFIR integrates multiple implementation theories into one overarching consolidated framework (Damschroder et al. 2009, 2022). The framework covers five main domains: “(1) the Innovation domain (i.e., the ‘thing’ being implemented, in this case YIM); (2) the Outer Setting domain (i.e., the setting in which the inner setting exists, in this case the juvenile justice system); (3) the Inner Setting domain (i.e., the setting in which the innovation is implemented, in this case Halt); (4) the Individuals domain (i.e., the roles and characteristics of individuals, such as the deliverers (professionals) and recipients (youths) of YIM); and (5) the Implementation Process domain (i.e. the activities and strategies used to implement the innovation).” View Table 3 of Damschroder et al. (2022). Applying CFIR offers the opportunity to identify standardised determinants that can be applied across multiple studies and contexts (Kirk et al. 2015).

## 3 Present Study

Successful implementation contributes to the effectiveness of an approach, but not much is known about the extent to which and how YIM is implemented. Therefore, we will explore the boosters and barriers (i.e., determinants) experienced by professionals in implementing YIM in the context of a short‐term diversion programme for justice‐involved youths. Specifically, we explored the following questions in a series of focus groups with professionals: (1) What factors facilitate the successful implementation of YIM (i.e., boosters)? and (2) What factors prevent the successful implementation of YIM (i.e., barriers)? Knowledge of these factors will contribute to implementation success, as they positively or negatively impact the actual use of an intervention (Fleuren et al. 2014).

## 4 Methods

The study was approved by the Ethics Review Board of the Faculty of Social and Behavioural Sciences at the Research Institute for Child Development and Education of the University of Amsterdam (#2022‐CDE‐14529). The current work follows the APA reporting guidelines for qualitative research (Qualitative research design JARS–Qual 2018).

### 4.1 Research Design Overview

We performed a series of focus groups to examine professionals' perspectives on implementing YIM. First, all participants took part in a large, unstructured focus group interview (*N*_total_ = 22) aimed at gaining an in‐depth understanding of the implementation of YIM to a previous youth case at Halt, and thereafter, participated in one of three smaller semi‐structured focus group interviews (*N* = 7–8) aimed at gaining insight into the boosters and barriers experienced in the implementation of YIM at Halt. For the semi‐structured focus groups, we developed a topic list based on the CFIR (Damschroder et al. 2009, 2022). We performed a multi‐step thematic approach (Braun and Clarke 2006). We applied inductive coding strategies (i.e. open coding) for the large focus group and deductive coding strategies (i.e., coding with a predefined set of codes) for the small focus groups.

We opted for focus group interviews for three main reasons. First, focus groups provide the opportunity to explore the perspectives of a population (i.e. Halt professionals trained in YIM) on a topic of interest (i.e., implementation of YIM) (Côté‐Arsenault and Morrison‐Beedy 2005; Tong et al. 2007). Second, focus groups resemble a more ‘natural’ conversation as compared to individual interviews (Krueger and Casey 2000; Överlien et al. 2005), which contributes to naturalistic responses on the topic of interest (Adams and Cox 2008). Lastly, focus groups, in contrast to individual interviews, help to reach and gain the perspective of a broader and more diverse sample of the population.

### 4.2 Implementation of YIM at Halt

To implement YIM at Halt, the Dutch YIM Foundation trained Halt professionals in each region throughout the Netherlands, namely North, North‐East, West and South. Professionals could actively choose to be trained in YIM. Approximately 20% of Halt professionals (*N* = 36) followed a 2.5‐day training, during which half a day was focused on translating the knowledge into their daily practice. The training focused on (1) the inspiration and motivation behind the importance of involving a mentor; (2) the five methodological YIM steps (*as described below*); and (3) developing skills to apply these steps, particularly how to respond effectively to varying responses from youth (e.g., “No, I do not know someone [to involve as a mentor]”). The training offers the foundation to initiate and monitor a YIM trajectory alongside the diversion programme.

In short, during the initial meeting with youths and their parents, professionals explain and motivate YIM (*step 1*). They ask youths the ‘YIM‐question’, which assesses *who* youths turn to aside from their parents. If youths are not able to name someone, professionals support youths by, for example, helping them visualise their social network. After identifying someone, parents are asked to give consent to the chosen YIM, after which the youths ask that person to be their mentor (*step 2*). Note that it is possible that youths are not able to nominate a YIM, but if a YIM is nominated, a ‘YIM‐conversation’ between the professional and the mentor follows (*step 3*), exploring the mentor's reaction to the youth's request and if and how they wish to engage. The professional inquires what the mentor needs to effectively take on the role, what they can and cannot do, and howthe professional can provide support to the mentor. This conversation aims to establish an equal partnership where each other's expertise is valued. Importantly, the mentor is not screened or trained by professionals. After this, the individual is officially ‘positioned’ as a YIM with all parties (youths, caregivers, mentors and professionals) present (*step 4*). Together, collaboration agreements are established (*step 5*), for example, on the youths' goals, the number of contact moments, and confidentiality. This impacts the intensity of the trajectory. If necessary, additional interim contact moments between the YIM and the professional take place. At the end of the diversion programme, approximately 3 months after referral, an evaluation meeting takes placeto assess the goals and determine whether to end the YIM trajectory. Note that a YIM can thus remain involved after finishing the diversion programme. Supervision is provided by Halt and the Dutch YIM Foundation to all professionals implementing YIM.

### 4.3 Participants

Twenty‐two YIM‐trained Halt professionals were approached face‐to‐face during a national YIM supervision meeting. This offered the opportunity to reach a diverse (i.e., professionals from all four regions) and a large majority of the population (i.e., 61.11% of all YIM‐trained professionals). The researchers verbally shared the aim and procedure of the research, after which professionals were provided with the consent form. All approached professionals (*N* = 22) agreed to participate. Before we started, we verified that each focus group had at least one professional from each region. This ensured diversity in the group's composition, which aids interaction and discussion (Côté‐Arsenault and Morrison‐Beedy 2005; Finch et al. 2014). No participants dropped out of the study.

The age of the participants ranged from 25 to 62 years (*M* = 36.09, SD = 9.96), and most participants were women (77.27%). Nineteen participants completed higher professional education (86.36%), and aside from being trained in YIM, 21 participants were also trained in motivational interviewing (95%). Professionals' experience with implementing YIM ranged from 1 to 3 years (*M* = 1.82, SD = 0.73). Large variations were observed in professionals’ years of working experience and years of working at Halt, respectively, 4 to 41 years (*M* = 12.64, SD = 9.52) and 2 to 29 years (*M* = 8.23, SD = 7.01). Half of the participants (50%) reported working at Halt for 5 years or fewer. Table 1 presents the demographics per focus group.

**Table 1:** Participant characteristics per focus group.

		**Focus groups**			
**Sample characteristics**		**Large**	**Small 1**	**Small 2**	**Small 3**
		**(** ***N*** = **22)**	**(** ***N*** = **7)**	**(** ***N*** = **7)**	**(** ***N*** = **8)**
**Age** in *years*	*M* (*SD*), Range	36.09 (9.96), 25–62	34 (9.93), 27–55	43.29 (10.03), 32–62	31.63 (6.93), 25–44
**Sex**, *N* (%)	Women	17 (77.27%)	7 (100%)	3 (42.86%)	7 (87.50%)
**Educational level**, *N* (%)	SVE	2 (9.09%)	1 (14.29%)	1 (14.29%)	0 (0%)
	HE	19 (86.36%)	5 (71.43%)	6 (85.71%)	8 (100%)
	UE	1 (4.55%)	1 (14.29%)	0 (0%)	0 (0%)
**Professional experience** in *years*	*M* (*SD*), range	12.64 (9.51), 4–41	11.86 (10.07), 4–32	18.71 (10.94), 11‐41	8 (4.47), 4–16
**Experience at Halt** in *years*	*M* (*SD*), range	8.23 (7.01), 2–29	4.43 (3.10), 2–11	13.29 (9.65), 5‐29	7.13 (4.22), 3–16
**Experience in YIM** in *years*	*M* (*SD*), range	1.82 (0.73), 1–3	1.28 (0.48), 1–2	2.28 (0.48), 2‐3	1.87 (0.83), 1‐3
**Region**, *N* (%)	North‐H‐C	6 (27.27%)	2 (28.57%)	3 (42.85%)	1 (12.5%)
	North‐E	5 (22.72%)	2 (28.57%)	1 (14.28%)	2 (25%)
	South	4 (18.18%)	2 (28.57%)	1 (14.28%)	1 (12.5%)
	West	7 (31.81%)	1 (14.28%)	2 (28.57%)	4 (50%)

Abbreviations: *HE* Higher Education; *North‐H‐C* North‐Holland‐Centre, *North‐E* North‐East; *SVE* Secondary Vocational Education; *UE* University Education.

### 4.4 Data Collection

Twenty‐two YIM‐trained Halt professionals, two Halt policy officers, a YIM trainer, a research assistant unfamiliar to the Halt professionals, and the main researcher (first author) were present during the data collection.

#### 4.4.1 Large Focus Group

We recreated a *reflecting team* model (Chang 2010; Van Dam 2007) with an inner circle (*N* = 6) and four smaller outer circles (*N*_per outer circle_ = 4) moderated by the YIM trainer and observed by the main researcher. In the inner circle, the trainer asked one professional to share a prior youth case in which the professional experienced difficulties implementing YIM. The other five professionals were instructed to help the trainer gain insight into the youth case and the implementation of YIM. The trainer used puppets to visualise the youths' social network. In the four outer circles, professionals were asked to observe what happened in the youth case regarding YIM from a specific perspective derived from the CFIR, namely the (1) youths, (2) caregivers, (3) professionals, or (4) the organisation's perspective (i.e. Halt) (Damschroder et al. 2009, 2022). Next, the inner and outer circles engaged in a joint discussion about the implementation of YIM in this youth case. The trainer started this discussion by asking professionals: “*Why did the implementation of YIM not succeed from the perspective of caregivers (or youths, professionals, or the organisation)*?” To increase the added value for professionals, they discussed a round of solutions for overcoming the challenges experienced with implementing YIM in this youth case. The duration of the focus group was 45 min.

Contrary to our published protocol (Boering et al. 2024), we used an unstructured format for the large focus group interview to allow us to gain an in‐depth understanding of the implementation of YIM in a specific case–a more tightly structured approach was considered to lead to the inhibition of responses (Zhang and Wildemuth 2017). Even though we adopted a structured task (i.e., sharing an unsuccessful YIM case, observing this from various perspectives and a starting question to the discussion), the YIM trainer followed the professionals' narratives (Zhang and Wildemuth 2017), consistent with how the YIM supervision meetings are usually moderated. This yielded a natural and familiar setting for the YIM‐trained professionals, contributing to feeling comfortable and, thus, encouraging more self‐disclosure (Côté‐Arsenault and Morrison‐Beedy 2005).

#### 4.4.2 Small Focus Groups

We performed three semi‐structured focus group interviews. The first was moderated by the main researcher and the research assistant, the second by the main researcher, and the third by the research assistant. The YIM trainer and Halt policy officers were not present. Based on three CFIR domains, namely the ‘Innovation, Individuals, and Inner Setting domains’, we developed a topic list tailored to the Halt context (see Table 2) (Damschroder et al. 2009, 2022). In the ‘Individuals’ domain, we focused on the characteristics of *deliverers* (i.e. Halt professionals) and *recipients* (i.e., youths) of YIM. Importantly, we did not ask questions regarding the ‘Outer setting’ and ‘Implementation process domains’ because the study primarily focused on the actual use of YIM by professionals *within* the organisation rather than the implementation efforts to initially implement YIM within this context. However, if the topic spontaneously came up, we did code this accordingly.

**Table 2:** Example questions of the topic list and key findings per CFIR domain.

CFIR domain	Example questions per domain	Key Findings: Perspectives of Professionals	
		Boosters	Barriers
**1. Innovation** The ‘thing’ being implemented (i.e., YIM)	What aspects do you find easy in the implementation of YIM? What aspects do you find complicated in the implementation of YIM?	**Relative advantage:** YIM is more durable and more accessible than solely Halt or referral to formal support systems. **Innovation adaptability:** It is possible to adapt YIM to the youths' needs.	**Innovation complexity:** Implementing YIM in addition to Halt required a larger time investment than solely implementing Halt.
**2. Inner setting** The setting in which the innovation is implemented (i.e., Halt)	How do you experience the support from the organisation to implement YIM? ◦ Training/coaching? ◦ Time? ◦ Support from management? In the ideal situation, what support would you need from Halt to properly implement YIM?	**Available resources: Materials and equipment:** Great variety of supporting tools and materials, easing implementation. **Access to knowledge and information:** Ample opportunities for support and coaching.	**Relative priority:** Implementing regular tasks of the diversion programme had relative priority over implementing YIM. **Structural characteristics:** The workload and great variety of tasks in the regular programme prevented the implementation of YIM. **Compatibility:** YIM's compatibility with Halt's workflow, systems and procedures impacts the decision not to implement YIM. **Incentive systems:** Taking the time to implement YIM could lead to unmet targets set by the organisation for regular tasks. **Mission alignment:** Some professionals did not experience the alignment of purpose and goals between YIM and Halt.
		**Mission alignment:** Some professionals experienced the alignment of purpose and goals between YIM and Halt.	
**3. Individuals** The roles and characteristics of individuals			
3.1 *Characteristics of deliverers* (i.e., Halt professionals)	How confident are you that you will be able to successfully implement the intervention?		
3.2 *Characteristics of recipients* (i.e., youths (and their parents))	Which factors in the youth case play a role in the successful implementation of YIM?	**Youths' needs, opportunity, and motivation:** The presence of risk factors, waiting lists or unsatisfied healthcare relationships, and the youth's motivation stimulate implementation.	**Youths' needs, opportunity, and motivation:** The absence of risk factors, the presence of too complex problems, and (strong) healthcare relationships prevent implementation.

In line with our published protocol (Boering et al. 2024), we performed semi‐structured interviews because they offered the opportunity to focus the interview on topics related to our research questions (Brinkmann 2020) and ensure consistency between focus group moderators. Moreover, to encourage individual participation, we activated professionals via *Think–Pair–Share* techniques (Kagan 1994; Lyman 1981). This cooperative learning technique enhances the productivity of discussions and improves the quality of responses (Kagan 1994). The duration of the focus groups was between 45 and 62 min and they were audio‐recorded and transcribed verbatim. If data saturation was not achieved after these focus groups, a fourth focus group with the remaining Halt professions would have been organised.

### 4.5 Analysis

We performed a multi‐step thematic analysis in MAXQDA software (Braun and Clarke 2006; Gizzi and Rädiker 2021) to identify boosters and barriers experienced by professionals in implementing YIM. We adopted both inductive and deductive coding strategies. First, for the large focus group, we developed an *initial coding framework* based on the perspectives of the CFIR (i.e. recipients and deliverers of YIM and the organisation). Due to the unstructured nature of this large focus group, the first author did not predefine a set of codes but employed open coding strategies, leading to an *initial codebook*. Second, for the smaller focus groups, we merged the initial codebook derived from the larger focus group into a codebook with a predefined set of codes linked to the domains of the CFIR relevant to our research questions (i.e. ‘Innovation, Individuals; *recipients and deliverers*, Inner Setting domains’) (Damschroder et al. 2022). This offered the opportunity to extract codes not covered by the framework. With this codebook, we applied deductive coding strategies to the small focus groups. The codebook was under constant development via an iterative process until no new codes were defined. Visual representations of the data, such as a code matrix browser and a summary matrix, aided the organisation of codes into boosters and barriers (Rädiker and Kuckartz 2020). Final themes were identified based on patterns of the most discussed topics.

#### 4.5.1 Quality Procedures

To ensure methodological integrity, we performed several quality procedures. Concerning the interviews, the topic list was reviewed by Halt policy officers as well as all authors. A 6‐h joint training stimulated consistency in the performance of moderators. Moreover, all transcripts were double‐checked for accuracy against the original audio files (Braun and Clarke 2006). Concerning the coding procedures, while the first author deployed all coding strategies, all authors were involved in constant discussion and revision of the codebook until a consensus was reached. Moreover, the final codebook was reviewed by the second (APG) and fourth author (GJO). Concerning the findings, we performed a member check by asking professionals for feedback to confirm that the findings were true to the professionals’ perspectives (Candela 2019). We followed several recommendations on how to apply the CFIR, such as reporting on the selection of CFIR domains and integrating the framework in all phases of our study (Kirk et al. 2015).

## 5 Results

Below, we describe boosters and barriers as perceived by professionals working with YIM in the context of a short‐term diversion programme. Boosters and barriers were discussed in all three focus groups unless otherwise indicated. Because we did not observe variations in domains between focus groups, we did not perform additional focus groups. Table 2 presents an overview of the findings.

### 5.1 Boosters

#### 5.1.1 Relative Advantage

Implementing YIM offered youths (and their families) more durable and accessible support than solely Halt or referral to formal support systems. Especially since formal healthcare professionals are only temporarily involved, and youths are quite often reluctant to receive formal care or are placed on waiting lists. A professional explained, “*Formal care very often has waiting lists, and a young person simply needs help now – whether it is practical or just needing a listening ear. Well, at least until more help is available, it is valuable to have your own network, someone you trust, to be there for you*.” Being able to choose their mentor facilitated the implementation of YIM. Overall, professionals perceived YIM as a valuable addition to the Halt programme, offering more options to prolong the support according to the youths' needs.

#### 5.1.2 Materials and Equipment and Access to Knowledge and Information

Professionals positively evaluated the materials and equipment and the facilitation of YIM. They had access to various supporting tools and materials – of good quality – which facilitated the introduction of YIM. Professionals felt they could easily contact colleagues and the YIM Foundation for support or advice. Moreover, they had access to individual coaching, regional intervision meetings, and national supervision meetings.

#### 5.1.3 Innovation Adaptability

In two out of three focus groups, professionals discussed being able to adapt the approach to youths' needs (Damschroder et al. 2022). For instance, a professional mentioned, “*You have room for creativity; you can make YIM your own. You can implement it lightly or heavier”*.

### 5.2 Barriers

#### 5.2.1 Relative Priority

Professionals indicated that implementing the regular tasks of the diversion programme at Halt was of relative priority to implementing YIM. Implementation of YIM was voluntary at Halt; thus, professionals opted to implement YIM or not. A professional disclosed, “*It is just because it's not yet part of Halt's regular duties. At least for me, then I'm more likely to put YIM aside.”* This decision was mainly impacted by other barriers, such as the organisation's structural characteristics and incentive systems, the approach's complexity and compatibility, and the youth's needs and motivation.

#### 5.2.2 Structural Characteristics: Work Infrastructure

The workload and the large variety of tasks at the organisation were perceived as a barrier to implementing YIM. About the workload, a professional stated, “*I notice in myself that if I have a youth case where I really think 'this youth needs a YIM', then I will simply implement it. But I have not offered it sufficiently to all youth cases because I thought if all those youths wanted a YIM, but they didn't really need it, I wouldn't be able to manage my caseload*.” About the variety of tasks, another professional added, “*To implement YIM in each youth case? The programme [Halt] is simply too big by itself. All the things you must do in the programme, and then YIM is extra.”*

#### 5.2.3 Innovation Complexity

Implementing YIM in a youth case required a larger time investment than solely implementing Halt. Most difficulties were experienced in the matching procedure, such as motivating youth for YIM or breaking through resistance and personal disappointment if a youth could not nominate someone. A professional explained, “*There was no one around her that she trusted. […] I was just disappointed that she did not have anyone in her environment*.” Moreover, after a match, keeping in contact with the mentor required more time from professionals, especially if the youth case at Halt was already ‘closed’ (*see* Compatibility). Three professionals stressed that these abovementioned experiences were due to their workload rather than the actual complexity and time investment of YIM, “*It's not really that the YIM is so much… It's more in combination with the other tasks*.”

#### 5.2.4 Compatibility

YIM is both compatible and less compatible with the workflow, systems, and procedures at Halt. However, because this clearly impacts the decision not to implement YIM, it is discussed as a barrier to implementation. Even though professionals in one focus group mentioned that investigating the social network is crucial to tailor the programme to the needs of the youth, professionals in two focus groups mentioned difficulties integrating YIM within their tasks. Specifically, both the regular Halt programme and YIM required more effort at the start, but YIM also required attention after finishing the regular programme. A professional indicated, “*How are you going to integrate this into the Halt programme? Especially the part where you close a youth case, but it continues for YIM… There are two tracks, and to what extent do youths and parents understand that Halt has finished but that there are still conversations [with the Halt professional]?”*

#### 5.2.5 Incentive Systems

Despite receiving extra time to implement YIM, taking the time for YIM could lead to unmet monthly targets set by the organisation. Even though professionals felt support and understanding from the organisation if targets were not met, this felt like a barrier to implementing YIM, “*And I know that I will not be punished for it, because if I have 20 youth cases with a YIM process… I can justify it, but still… You know, if you get an email [with your and your team's monthly targets for the regular tasks] and you see your name in red, then you think, “I'm in red again*”. However, two other professionals mentioned they did not experience this barrier and took the time to implement YIM if necessary.

### 5.3 Factors That Could Contribute as a Booster or Barrier

#### 5.3.1 Mission Alignment

Some professionals experienced the alignment of purpose and goals between YIM and Halt, while others did not. For some professionals, YIM was the perfect tool for Halt, “*I was searching for an opportunity to follow youths a bit longer at Halt. It's a short‐term intervention, and sometimes more help is necessary, but we did not have this option. Then YIM came along… the perfect tool to do so!*” Others doubt whether YIM was aligned with the mission at Halt, “*I have a legal background, and sometimes I encounter too much youth care, and the contact is too long; how far do you go as Halt? What is your role then?*” They believed that YIM's long‐term character, which has roots in youth care, does not align with Halt's short‐term character, which has roots in legal justice. Thus, an individual's perspective on the mission alignment had an impact on the choice to implement YIM.

#### 5.3.2 Youths' Needs, Opportunity and Motivation

Professionals considered a variety of youth case factors, including risk factors, social networks, the dynamic between YIM and formal care, and youth motivation, when deciding whether to implement YIM. Generally, the presence of risk factors stimulated them to implement YIM. It is thought that youths specifically benefited from YIM in the presence of specific risk factors. Professionals mentioned school absenteeism, tensions and conflicts in the home situation, single parenthood, a closed family system (i.e. families that are more inward‐focused and less open towards external influences (Wirick and Teufel‐Prida 2019), seemingly making them more receptive towards involving a YIM from their social network), absence of a social network, lower socioeconomic status, and cultural differences. A professional explained, “*There is no need to implement it [YIM] in ‘light’ [no to low risk] youth cases. Only as soon as you, of course, identify risks when you start the conversation, yes, then you should have the gut feeling, here it is appropriate [to implement YIM]*”. However, if problems were too complex, professionals believed that formal healthcare was a more suitable option than YIM. The dynamic between youths and formal healthcare impacts the opportunity to implement YIM. Professionals felt reluctant to implement YIM if youths had (strong) formal healthcare relationships and felt encouraged if youths were on the waiting list or were reluctant towards or unsatisfied with formal healthcare relationships. Notably, the youth's (and their families) motivation for working with YIM was crucial for successful implementation and impacted the professionals’ motivation to implement YIM.

## 6 Discussion

With our study, we aimed to examine what factors contribute to (un)successful implementation of YIM, as perceived by professionals implementing YIM as an addition to a short‐term diversion programme for justice‐involved youths. Boosters we identified were that, in the eyes of professionals, YIM offers more accessible and more durable support than implementing Halt alone or referring youths to formal care, that YIM could be adapted to the needs of the youth, and that the facilitation of YIM was good (e.g. suitable materials and ample opportunities for support). Barriers we identified were that Halt professionals prioritised the regular programme over implementing YIM due to the heavy caseload, the great variety of tasks in the programme, and the emphasis on the assessment of performing the regular (Halt) tasks by the organisation. Specific to YIM and its compatibility with the Halt programme, a larger time investment and difficulties were experienced in the matching procedure and during match support (i.e. supporting youths, parents, and mentors in building their relationships). Our study also identified two factors that could be considered both a booster and a barrier to implementation: mission (mis)alignment between YIM and a variety of youth case factors, such as risk factors and youths' motivation for YIM.

In this selective prevention context, successful implementation was strongly guided by the RNR model (Bonta and Andrews 2007). In line with the *risk* and *needs* principles, professionals sought a balance for whom it was suitable to implement YIM. They argued that YIM is suitable for youth who need support and are specifically inclined to implement YIM in the presence of (any type of) risk factors, such as school absenteeism or with tensions and conflicts in the home situation. However, in the absence of risks, professionals did not perceive the added value and were quick to proceed with a minimal regular programme, and in the presence of high risks, they preferred referring youths to formal support structures. Professionals carefully consider the level of *risk* and the youths' *needs* and, thus, play an essential role in the successful implementation of YIM within this context. This contrasts with the YIM program theory that YIM is suitable for youth with varying levels of risk factors across different contexts: universal prevention, selective prevention, and indicated prevention (Mrazek and Haggerty 1994; Van Dam and Schwartz 2020), as well as prior findings that suggest that YIM is suitable for all youth regardless of risk status (Van Dam et al. 2018). Aside from the actual implementation, this misalignment could potentially endanger YIM's sustainability – the continuation of an innovation – over time (Wiltsey Stirman et al. 2012). Thus, organisations working according to the RNR principles that aim to implement YIM should be aware of this suboptimal alignment with YIM and make an informed and proactive decision about implementation strategies related to the risks of the youths, as well as strategies that are critical to sustainability over time (Wiltsey Stirman et al. 2012). For example, an organisation should make sure if and how it is possible to align the innovation's mission with the existing organisational culture. Together with professionals implementing an RNR intervention with YIM, they should critically evaluate when to implement YIM, for *whom*, and whether it is possible to tailor the scope and intensity of YIM to match the youth's risk level and needs. To facilitate the extent to which such decisions can be well‐informed, future research should gain insight into the benefits of implementing YIM for youths with varying risk factors across different contexts and develop practical guidelines for *whom* YIM is suitable. A more fundamental change is directed at reconsidering the central cultural principles and arriving at a ‘cultural shift’ within professionals and the organisation.

Implementing YIM as an add‐on to a short‐term (diversion) programme could potentially increase effective support, specifically for vulnerable youths. Professionals indicated that YIM offered them the opportunity to monitor these youths for a longer period. Moreover, professionals felt they actively contributed to the youths' future by anchoring the support in the youths' social network – something more durable after their involvement had ended. Even though, in this study, it remains unknown how long the mentor‐mentee matches lasted, we can speculate that once these relationships are established, they are durable (Spencer et al. 2016, 2021; Dam et al. 2017), which has been linked to an increased likelihood of positive youth outcomes (Schwartz et al. 2013; Grossman and Rhodes 2002; Spencer et al. 2016). Potentially, it could also address the erosion effects of the short‐term programme after professional involvement has ended (Schwartz et al. 2013). However, prolonging the programme is at odds with Halt's legal context and vision to ensure the programme is completed as quickly as possible. Consequently, this causes tension in practice between the perceived advantages and the available time to implement YIM properly, impacting the actual and possibly sustained implementation of YIM. Future research should focus on the durability of the mentor‐mentee relationships in this population and the (dis)advantages of implementing YIM within a short‐term programme.

If one aims to adopt YIM as an add‐on to a short‐term (diversion) programme, our findings suggest it is essential to focus on both the matching procedure and the match support of YIM. Difficulties with implementation were mainly experienced in the YIM matching procedure. They were similar to professionals' perspectives in prior research (Spencer et al. 2021), such as motivating youth or breaking through resistance for YIM, identifying a mentor, and the time to implement YIM. Therefore, it is crucial to focus training and coaching on these difficulties. For example, how to navigate resistance and what to do if youths are not able to identify a mentor (e.g. how to deal with a youth's (or own) disappointment, or to offer alternative options, such as matching youth with a formal mentor). Even though professionals in prior research perceived match support to be less intensive compared to formal mentoring models (Spencer et al. 2021), professionals at Halt perceived their role in match support as relatively intensive. It required them to stay in contact longer after regular activities were finished, increasing their workload. These difficulties and the continuously larger time investment require careful consideration of how to adapt and embed YIM into the organisation's workflow, or to give professionals proper incentives to implement the innovation. For example, a critical assessment should be performed at the organisational level of how to integrate key components of YIM into core elements of the intervention or possibly omit certain elements of the existing intervention, to explicitly allocate time to implement YIM. Moreover, one could make clear agreements about the professional's involvement after the closure of the intervention, or make sure the caseload is temporarily decreased so professionals can allocate time to the match support after the diversion programme has ended. Making optimal adaptations to embed YIM into the professionals' daily tasks should contribute to professionals' motivation to implement YIM, but it is also important if one aims to sustain the implementation of YIM over time. Although Halt professionals consider the adaptability of YIM a facilitating factor for implementation, future research can teach us more about which core elements of YIM are effective, making it possible to adapt and sustain YIM more effectively into a short‐term programme (or into other contexts).

The current study has several strengths. This study is among the first to gain insight into how professionals experience the implementation of YIM. Specifically, we used a theoretical framework (i.e. CFIR) to identify boosters and barriers in a juvenile diversion programme, which provides the opportunity to adapt and apply these standardised determinants to other contexts (Damschroder et al. 2022). The findings also provide insights into factors that impact the actual and possibly sustained implementation of an innovation at an organisation, which is something fewer studies have focused on (Wiltsey Stirman et al. 2012). Moreover, we were able to reach a large majority of all YIM‐trained Halt professionals (71%), ensuring that our findings accurately reflect the population of interest.

Several study limitations warrant mentioning as well. Firstly, we asked professionals to take the youths' perspectives (and their caregivers') instead of directly targeting these individuals. Although we believe that perspective‐taking gives insight into factors that facilitate or negate the implementation of YIM by professionals, we also think that future research should focus on the actual perspectives and experiences of youths, caregivers, and YIMs in the juvenile delinquency field. Secondly, the pre‐existing researcher‐participant relationship (i.e. working together for over a year in the context of an effectiveness study of YIM at Halt (Boering et al. 2024)) could potentially limit the participants' ease of discussing all types of attitudes towards YIM. Moreover, participants might infer that the researcher is already familiar with the answers (McDermid et al. 2014). However, no notable differences were observed in positive or negative attitudes towards YIM, nor in how often participants referred to the effectiveness study between the focus groups moderated by the familiar or unfamiliar researcher. Nevertheless, future research should consider the possible impact of a pre‐existing researcher‐participant relationship. Thirdly, performing all focus groups on the same day has possibly led to transfer effects from the large to the smaller focus groups. Specifically, when evaluating YIM in the smaller focus groups, we observed that – in some cases – professionals referred to the unsuccessful youth case discussed in the large focus group, possibly contributing to perceiving more barriers to YIM. If several focus groups are performed in future research, researchers should consider the time and order between the focus groups.

This study highlights the importance of examining boosters and barriers to the implementation of new methodologies and/or practices in judicial settings or selective prevention contexts, specifically because these factors impact actual and effective implementation. Our findings demonstrate that professionals perceive and believe in the benefits of YIM in their short‐term diversion programme, but that some characteristics of the current organisational culture (i.e., being strongly grounded in a *Risks‐Needs‐Responsivity* (RNR) framework) hindered the actual implementation of YIM. This misalignment between the organisational values and the YIM program theory possibly endangers the continuation of the implementation of YIM as intended. Future organisations implementing the YIM approach should be aware of this delicate balance between their organisational values and the values of this new approach.

## Author Contributions

Funding for this study was acquired by Levi van Dam. All authors contributed to the study conception and design. Material preparation, data collection, coding, and analyses of the data were performed by Angelique Boering in close consultation with and supervision of all authors. The first draft of the manuscript was written by Angelique Boering and all authors commented on previous versions of the manuscript. All authors read and approved the final manuscript.

## Ethics Statement

This study is being conducted in compliance with the Declaration of Helsinki and was approved by the Ethics Review Board of the Faculty of Social and Behavioural Sciences at the Research Institute for Child Development and Education of the University of Amsterdam (#2022‐CDE‐14529). Participants were informed about the study's procedure and required to give consent before participation.

## Conflicts of Interest

Levi van Dam developed an educational programme for professionals to learn about the scientific and practical background of youth‐initiated mentoring. The other authors declare no conflicts of interest.

## Peer Review

The peer review history for this article is available at https://www.webofscience.com/api/gateway/wos/peer‐review/10.1002/jcop.70051.

## Data Availability

Due to the nature of the current data, the datasets generated and/or analysed are not publicly available due to privacy or ethical restrictions. Still, data are available from the corresponding author upon reasonable request via a secured access repository.
